# Cost-effectiveness analysis based on two models of sacituzumab tirumotecan versus platinum-based chemotherapy as second-line treatment in EGFR-mutant non-small-cell lung cancer

**DOI:** 10.3389/fpubh.2026.1802471

**Published:** 2026-05-07

**Authors:** Lijun Jin, Lu Han, Jing Zeng

**Affiliations:** 1 Department of Pharmacy, Wenzhou People’s Hospital, Wenzhou, China; 2 Department of Pharmacy, Affiliated Jiangning Hospital of Chinese Medicine, China Pharmaceutical University, Nanjing, China

**Keywords:** cost-effectiveness analysis, Markov model, non-small cell lung cancer, partitioned survival model, sacituzumab tirumotecan

## Abstract

**Background:**

For patients with advanced EGFR-mutant non-small cell lung cancer (NSCLC) who progress after first-line therapy, effective subsequent options are urgently needed. From the perspective of China’s healthcare system, this study evaluated the cost-effectiveness of sacituzumab tirumotecan (sac-TMT) versus conventional platinum-based chemotherapy, using both a partitioned survival model (PSM) and a Markov model.

**Methods:**

Clinical efficacy and safety data were derived from the OptiTROP-Lung04 trial. Key health economic outcomes included direct medical costs, quality-adjusted life years (QALYs), and the incremental cost-effectiveness ratio (ICER). Model robustness was tested via one-way and probabilistic sensitivity analyses (OWSA, PSA), plus scenario analysis.

**Results:**

In the PSM, sac-TMT incurred an incremental cost of $64,948.58 and a gain of 0.74 QALYs versus chemotherapy, yielding an ICER of $87,922.33/QALY. The Markov model showed similar trends: an incremental cost of $52,075.41 for 0.67 QALYs, with an ICER $78,131.64/QALY—both far above the willingness-to-pay threshold (3 × 2025 GDP per capita). OWSA identified key drivers (e.g., progression free survival utility, progressive disease utility, sac-TMT price, the incidence of neutropenia in the sac-TMT arm), and base-case results remained stable within reasonable ranges. Notably, PSA revealed a 0% probability of sac-TMT being cost-effective under the current threshold. Scenario analysis indicated that including sac-TMT in medical insurance or raising the payment threshold could make cost-effectiveness feasible (up to 100% probability).

**Conclusion:**

In China’s healthcare setting, sac-TMT is not cost-effective for second line treatment of advanced EGFR-mutant NSCLC compared with platinum-based chemotherapy. To improve patient access, substantial price reductions or inclusion in the National Reimbursement Drug List are necessary. These findings may inform both clinical decisions and health resource allocation.

## Introduction

Lung cancer consistently holds a dominant position in terms of both high incidence and high mortality among global malignancies, with non-small cell lung cancer (NSCLC) accounting for over 85% of all cases (1). Among Chinese patients with NSCLC, epidermal growth factor receptor (EGFR) mutation is the most prevalent oncogenic driver, which provides the molecular basis for targeted therapy (2, 3). Although third-generation EGFR-tyrosine kinase inhibitors (TKIs) have become the standard first-line treatment for advanced EGFR-mutant NSCLC, acquired resistance and subsequent disease progression remain largely unavoidable in most patients (4). Currently, platinum-based doublet chemotherapy remains the main second-line strategy after EGFR-TKI failure; however, its survival benefit is modest and falls far short of meeting the clinical demand for more effective therapies (5, 6).

A novel antibody-drug conjugate (ADC), sacituzumab tirumotecan (sac-TMT), targets human trophoblast cell-surface antigen 2 (Trop-2). Trop-2 is widely expressed in EGFR-mutant NSCLC, and its overexpression correlates with resistance to EGFR-TKIs (7). Notably, preclinical evidence demonstrates that EGFR mutations promote the intracellular accumulation of sac-TMT, resulting in significantly higher drug levels in TKI-resistant cells compared with TKI-naive counterparts (6).

The pivotal OptiTROP-Lung04 trial (NCT05870319) was an open-label, randomized, multicenter phase III study that compared sac-TMT with platinum-based chemotherapy in patients with locally advanced or metastatic EGFR-mutant NSCLC who had progressed on prior EGFR -TKI therapy. Results showed that sac-TMT yielded a median progression-free survival (PFS) of 8.4 months, representing a significant improvement over chemotherapy and a 51% reduction in the risk of progression disease (PD) or death [hazard ratio (HR) for PFS, 0.49]. Notably, while the median overall survival (OS) in the chemotherapy arm was 17.4 months, the median OS in the sac-TMT arm had not yet been reached, corresponding to a significant 40% reduction in mortality risk (HR for OS, 0.60). Sac-TMT also demonstrated superior objective response rates (60.6% vs. 43.1%) and a longer median duration of response (8.3 vs. 4.2 months). The safety profile of sac-TMT was manageable, with a comparable incidence of grade ≥3 treatment-related adverse events (TRAEs) but a significantly lower rate of serious TRAEs (9.0% vs. 17.6%) compared with chemotherapy (4). Consequently, sac-TMT has been approved and established as a new standard second-line therapy. However, its cost-effectiveness within the Chinese healthcare system remains unclear. Therefore, from the Chinese healthcare system perspective, we performed a cost-effectiveness analysis of sac-TMT versus platinum-based chemotherapy using a partitioned survival model (PSM) and a Markov model, aiming to provide evidence for clinical practice and healthcare policy making.

## Methods

### Population and interventions

The modeled patient cohort was defined based on the key inclusion criteria of the OptiTROP-Lung04 trial. Key inclusion criteria were: (1) age 18–75 years; (2) histologically or cytologically confirmed locally advanced (stage IIIB/IIIC) or metastatic (stage IV) non-squamous NSCLC, ineligible for curative surgery or defi nitive chemoradiotherapy; (3) confirmed EGFR-sensitive mutation; (4) documented disease progression following prior EGFR-TKI therapy (progression after first−/second-generation TKI with confirmed T790M negativity, or progression after third generation TKI); (5) Eastern Cooperative Oncology Group (ECOG) performance status of 0 or 1, with a life expectancy of ≥12 weeks.

A total of 376 patients from the OptiTROP-Lung04 trial were randomized (1:1) to receive either sac-TMT or platinum-based chemotherapy. In the sac-TMT arm, sac-TMT was administered intravenously at a dose of 5 mg/kg on days 1 and 15 of each 28-day cycle. In the chemotherapy arm, patients received pemetrexed (500 mg/m^2^) combined with either carboplatin (target *AUC* 5 mg/mL·min) or cisplatin (75 mg/m^2^) on day 1 of each 21-day cycle. Following four induction cycles, patients in the chemotherapy arm without disease progression received pemetrexed maintenance therapy. Treatment continued in both arms until disease progression, unacceptable toxicity, withdrawal of consent, or physician’s decision. For the post-progression phase of the model, subsequent treatment strategies were based on the trial protocol [4] and contemporary Chinese clinical guidelines (5). It was assumed that all patients received subsequent therapy, which could include pemetrexed, osimertinib, or best supportive care, until death.

### Model construction

**Figure 1:**
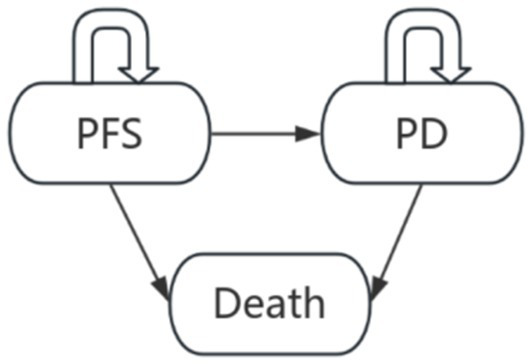
Three-state PartSA model and Markov model.

A PSM and a Markov model were constructed using TreeAge Pro software (Figure.1). Patients were classified into three health states: PFS, PD, and death. The model assumed that all patients entered the model in the PFS state, could transition to PD, and ultimately progress to death. In each cycle, patients occupied only one health state at a time, and received corresponding treatment regimens according to their state. In the Markov model, transition probabilities between states were estimated from survival curve parameters, with a half-cycle correction applied (8). In the PSM, the proportion of patients in each health state was determined by the areas under the PFS and OS curves. The model adopted a 10-year time horizon, sufficient to capture over 99% of expected mortality within the cohort. In accordance with the China Guidelines for Pharmacoeconomic Evaluations (2020), both future costs and health outcomes were discounted at an annual rate of 5%. Cost-effectiveness was assessed using the ICER. The willingness-to-pay (WTP) threshold was set at three times China’s per capita gross domestic product (GDP) for the year 2025. Using data published by the National Bureau of Statistics of China (8), the WTP threshold was calculated as US dollars 41,811 per quality-adjusted life year (QALY). The primary outcomes of this study were total costs, the incremental cost-effectiveness ratio (ICER), and QALYs.

### Clinical data input

Individual patient-level time-to-event data for OS and PFS were reconstructed by digitizing the published Kaplan–Meier curves from the OptiTROP-Lung04 trial using Engauge Digitizer software. The digitized coordinates were processed using R (version 4.4.2) to generate reconstructed individual patient data (IPD). The accuracy of the reconstruction was validated through visual and statistical comparison against the original published curves (Supplementary Table S1 and Supplementary Figure S1). To extrapolate survival beyond the trial observation period, the reconstructed IPD were fitted to seven candidate parametric survival distributions: Exponential, Weibull, Gompertz, Log-logistic, Log-normal, Gamma, and Generalized Gamma. The optimal distribution for each endpoint (OS and PFS) within each treatment arm was selected based on the lowest Akaike Information Criterion (AIC) and Bayesian Information Criterion (BIC) values, complemented by a visual assessment of the goodness of fit against the Kaplan–Meier estimates (9). The AIC and BIC values for all candidate distributions are provided in Supplementary Table S2, and the scale and shape parameters of the selected optimal distributions are presented in Table 1.

**Table 1:** Key model inputs.

Parameters	Baseline value	Low	High	Distribution	Source
Survival model of Sac-TMT					
Log-logistic model for OS	μ/(γ) = 1.538; σ/(λ) = 27.354			—	
Log-normal model for PFS	μ/(γ) = 2.20; σ/(λ) = 0.999			—	
Survival model of chemotherapy					
Weibull(PH) model for OS	μ/(γ) = 1.599; σ/(λ) = 0.007			—	
Log-logistic model for PFS	μ/(γ) = 2.420; σ/(λ) = 4.590			—	
Cost per cycle ($)					
Sacituzumab tirumotecan for injection (200 mg)	1,315.85	1,052.68	1,579.02	Gamma	Yaozh.com
Pemetrexed disodium for injection (500 mg)	65.52	52.42	78.62	Gamma	
Carboplatin injection (15 mL:150 mg)	11.06	8.85	13.27	Gamma	
Cisplatin for injection (200 mg)	1.71	1.37	2.05	Gamma	
Osimertinib mesylate tablets (80 mg)	23.18	18.54	27.81	Gamma	
Best supportive care (per cycle)	436.19	348.95	523.43	Gamma	(14)
Follow-up procedure costs					
Complete blood count/per test	2.10	1.68	2.52	Gamma	Zhejiang Provincial catalog^1^
Blood biochemistry/per test	25.20	20.16	30.24	Gamma	
Urinalysis/per test	2.52	2.02	3.02	Gamma	
CT scan/per exam	11.20	8.96	13.44	Gamma	
MRI scan/per exam	70.00	56.00	84.00	Gamma	
Nursing fee/per day	3.64	2.91	4.37	Gamma	
Bed fee/per day	2.80	2.24	3.36	Gamma	
Consultation fee/per visit	3.08	2.46	3.70	Gamma	
Intravenous injection fee/per administration	0.81	0.65	0.97	Gamma	
Antineoplastic chemotherapy drug preparation/per time	4.76	3.81	5.71	Gamma	
AEs cost					
Anemia	448.56	358.85	538.27	Gamma	(15)
Leukopenia	421.40	337.12	505.68	Gamma	(16)
Neutropenia	406.56	325.25	487.87	Gamma	(15)
Thrombocytopenia	2,937.46	2,349.97	3,524.95	Gamma	(15)
Utility values					
PFS	0.804	0.536	0.840	Beta	(14,18)
PD	0.321	0.031	0.473	Beta	
Disutility of AEs (per cycle)					
Anemia	−0.073	−0.088	−0.058	Beta	(14,18)
Leukopenia	−0.200	− 0.240	−0.160	Beta	
Neutropenia	−0.200	−0.240	− 0.160	Beta	
Thrombocytopenia	−0.190	−0.228	− 0.152	Beta	
AE incidence-sac-TMT arm					
Anemia	0.110	0.088	0.132	Beta	(4)
Leukopenia	0.280	0.224	0.336	Beta	
Neutropenia	0.400	0.320	0.480	Beta	
Thrombocytopenia	0.020	0.016	0.024	Beta	
AE incidence-chemotherapy arm					
Anemia	0.140	0.112	0.168	Beta	(4)
Leukopenia	0.220	0.176	0.264	Beta	
Neutropenia	0.330	0.264	0.396	Beta	
Thrombocytopenia	0.160	0.128	0.192	Beta	

^1^ Costs sourced from the Zhejiang Provincial Basic Medical Insurance Service Item Catalogue, considered representative of national pricing.

^2^ All costs were calculated in 2025 US dollars (USD) and discounted at an annual rate of 5% in the base-case analysis. All parameters were varied by ±20% from the baseline values, except for PFS and PD, which were directly derived from the 95% confidence intervals reported in the literature.

^3^ For probabilistic sensitivity analysis, Gamma distributions were used for costs and Beta distributions for utilities and probabilities. The incidence of thrombocytopenia in the sac-TMT arm was fixed (range equal to baseline) due to low observed variance in the source data.

In the Markov model, the disease progresses through multiple mutually exclusive health states with defined transition probabilities. These transition probabilities were time-dependent and could vary across model cycles. Transition probabilities were calculated following the method described by Zhou et al. (10). Specifically, the transition probability from the PFS state to death was set equal to the natural mortality rate. Given a model cycle length of 28 days, the annual natural mortality rate was converted into a 28-day probability using the method proposed by Gidwani et al. (11). The annual natural mortality rate was derived from the 2025 data released by the National Bureau of Statistics of China, which reported a crude mortality rate of 8.04‰ (8).

### Cost and utility input

Consistent with the Chinese healthcare system perspective, only direct medical costs were considered. These included drug acquisition costs, best supportive care, management of grade ≥3 adverse events, and routine follow-up procedures (including laboratory tests, imaging examinations, and hospitalizations). To maintain model parsimony, only severe TRAEs (grade ≥3) with an incidence of ≥5% in the OptiTROP-Lung04 trial were included in the cost calculation (4). Drug prices were based on the national median tender prices in China between January and December 2025 (12). The costs of follow-up procedures were obtained from the publicly available price list of the Zhejiang Provincial Basic Medical Insurance Service Item Catalogue, which is representative of national pricing standards. Costs for best supportive care and AE management were derived from Chinese published literature (13–15). All costs were adjusted to 2025 US dollars using the annual inflation rate (data sourced from https://www.inflationtool.com), and all Renminbi (RMB) costs were converted to US dollars using the 2025 annual average exchange rate of 7.1429 RMB per 1 USD. To estimate patient-level resource use, standard patient characteristics were applied: a body weight of 65 kg, a body surface area of 1.72 m^2^, and a creatinine clearance rate of 70 mL/min (16). Health state utility values for PFS and PD, along with disutility weights for severe TRAEs, were sourced from published utility studies in NSCLC populations (17). All model parameters, including base-case values, ranges for sensitivity analyses, and data sources, are summarized (Table 1).

### Sensitivity and uncertainty analyses

To evaluate the robustness of the base-case findings and characterize parameter uncertainty, deterministic one-way sensitivity analysis (OWSA) and probabilistic sensitivity analysis (PSA) were performed. In the OWSA, each key model parameter was varied independently within a plausible range. As recommended by guidelines for scenario exploration (18), the discount rate was tested from 0 to 8%, while all other parameters were varied by ±20% from their baseline values or 95% of confidence intervals were used as the basis for the range of uncertainty for each parameter. The impact of these variations on the ICER is presented in a tornado diagram. For the PSA, parameter uncertainty was propagated simultaneously using a second-order Monte Carlo simulation with 1,000 iterations. Consistent with established methodological practices, cost parameters were assigned Gamma distributions, while utility, disutility, and probability parameters (e.g., the incidence of adverse events) were assigned Beta distributions. The results of the PSA are visualized in an incremental cost effectiveness scatterplot and a cost-effectiveness acceptability curve (CEAC).

### Scenario analysis

#### Scenario 1: anticipated medical insurance reimbursement

To effectively improve the clinical accessibility and affordability of innovative anticancer drugs, China has implemented an annual dynamic adjustment mechanism for the National Reimbursement Drug List (NRDL), with continuous incorporation of eligible novel antineoplastic agents into the medical insurance system. According to the latest NRDL, carboplatin and cisplatin are classified as Category A drugs, while pemetrexed, osimertinib, and sac-TMT are listed as Category B drugs. Notably, the newly approved indication of sac-TMT based on the positive results of the OptiTROP-Lung04 study—was not included in the current NRDL negotiation process, as its approval date fell after the formal review deadline for the 2025 negotiation cycle. However, the two previously approved indications of this drug have been successfully included in Category B of the NRDL, with reimbursement based on the negotiated price effective January 1, 2026. Although the inclusion of this specific indication into the NRDL appears to be an inevitable trend, its current exclusion still imposes a considerable economic burden on patients. Therefore, this study conducted a cost-effectiveness analysis of the sac-TMT regimen under the scenario where this indication is not yet covered by medical insurance, using the NRDL-negotiated price ($637 per 200 mg) as the reference for cost calculation.

#### Scenario 2: impact of different payment thresholds

Health payment thresholds are used to define the maximum cost that society is willing to bear to obtain additional health gains, thereby judging whether a new drug intervention is cost-effective and achieves “value for money,” and further optimizing the allocation efficiency of limited medical insurance resources. Different thresholds collectively form a continuous decision-making spectrum from strict to loose, promoting the transformation of drug evaluation from merely “clinically effective” to balancing “economic affordability and social acceptability,” maximizing the efficiency of health resource allocation, reducing decision subjectivity, and improving the transparency and scientificity of health policy formulation. In this study, the differences in cost-effectiveness of the sac-TMT treatment regimen were analyzed when the WTP threshold was set at 1-time, 2-time, and 3-time the per capita GDP, respectively.

#### Scenario 3: regional variation in WTP thresholds

In this study, the WTP threshold was set at three times the 2025 per capita GDP of Beijing, Shanghai, Jiangsu, and Zhejiang. These regions serve as key pillars supporting China’s national medical insurance fund, and the relevant findings provide critical references for National Reimbursement Drug List negotiations. This scenario analysis establishes a “most optimistic economic assumption” to test the robustness of the study conclusions in regions with high payment capacity. If the ICER exceeds the above threshold, it suggests that the study conclusions are relatively robust. Conversely, if the ICER falls below the threshold, further exploration is warranted regarding disparities in drug accessibility across regions with different economic levels. Meanwhile, this scenario also indirectly assesses whether drugs that are not cost-effective at current prices could achieve limited clinical accessibility in economically developed regions through strategies such as regional pricing or risk-sharing agreements.

## Results

### Base-case analysis results of the two models

In the PartSA model, the total cost for the sac-TMT group was $73,879.31, yielding 1.32 QALYs, while the total cost for the chemotherapy group was $8,930.73, yielding 0.59 QALYs. The ICER for sac-TMT versus chemotherapy was $87,922.33/QALY; In the Markov model, the base-case analysis demonstrated that sac-TMT provided an incremental health benefit of 0.67 QALYs compared to platinum-based chemotherapy, at an additional cost of $52,075.41. The corresponding ICER was $78,131.64 per QALY gained. This ICER substantially exceeded the prespecified WTP threshold of three times China’s 2025 per capita GDP ($41,811 per QALY). Consequently, at current pricing, sac-TMT is not a cost-effective treatment option versus chemotherapy for patients with EGFR-mutant NSCLC in the Chinese healthcare setting (Table 2).

**Table 2:** Base-case analysis results of the two models.

Model	Treatment	Total cost	∆ Cost	QALYs	∆ QALYs	ICER ($/QALY)
PSM	Chemotherapy	8,930.73	—	0.59	—	—
	sac-TMT	73,879.31	64,948.58	1.32	0.74	87,922.33
Markov	Chemotherapy	10,715.65	—	0.60	—	—
	sac-TMT	62,791.06	52,075.41	1.26	0.67	78,131.64

This table summarizes the total costs, QALYs, incremental outcomes and iICERs for the two treatment strategies. All costs are reported in US dollars (USD).

### One-way sensitivity analysis

**Figure 2:**
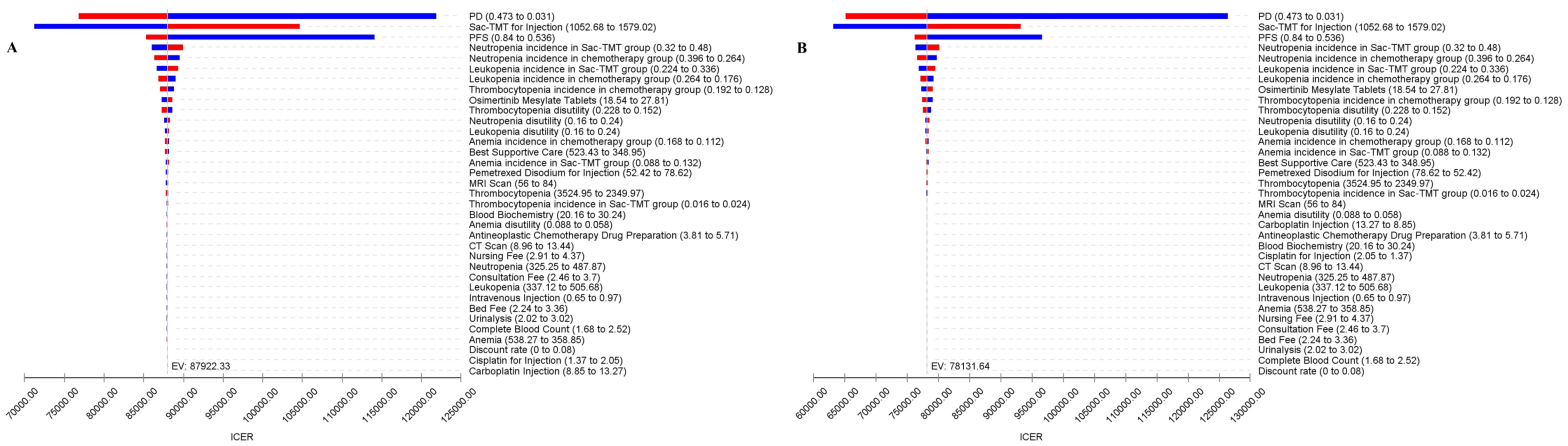
One-way sensitivity analysis. **(A)** PSM. **(B)** Markov model. Based on the tornado diagram, key parameters influencing the incremental cost-effectiveness ratio (ICER, USD/QALY) of sac-TMT versus platinum-based chemotherapy in the base-case analysis are presented. Each horizontal bar represents the variation in the ICER when a specified parameter fluctuates across its predefined plausible range.

The tornado diagram (Figure 2) showed that the key parameters influencing the ICER were similar between the two models. The cost of sac-TMT, PFS, PD, and the incidence of neutropenia in the sac-TMT arm each had a substantial impact on the ICER. Across a range of plausible parameter variations, the ICER consistently exceeded the WTP threshold of $41,811 per QALY, indicating that sac-TMT was not cost-effective compared with chemotherapy.

### Probabilistic sensitivity analysis

**Figure 3:**
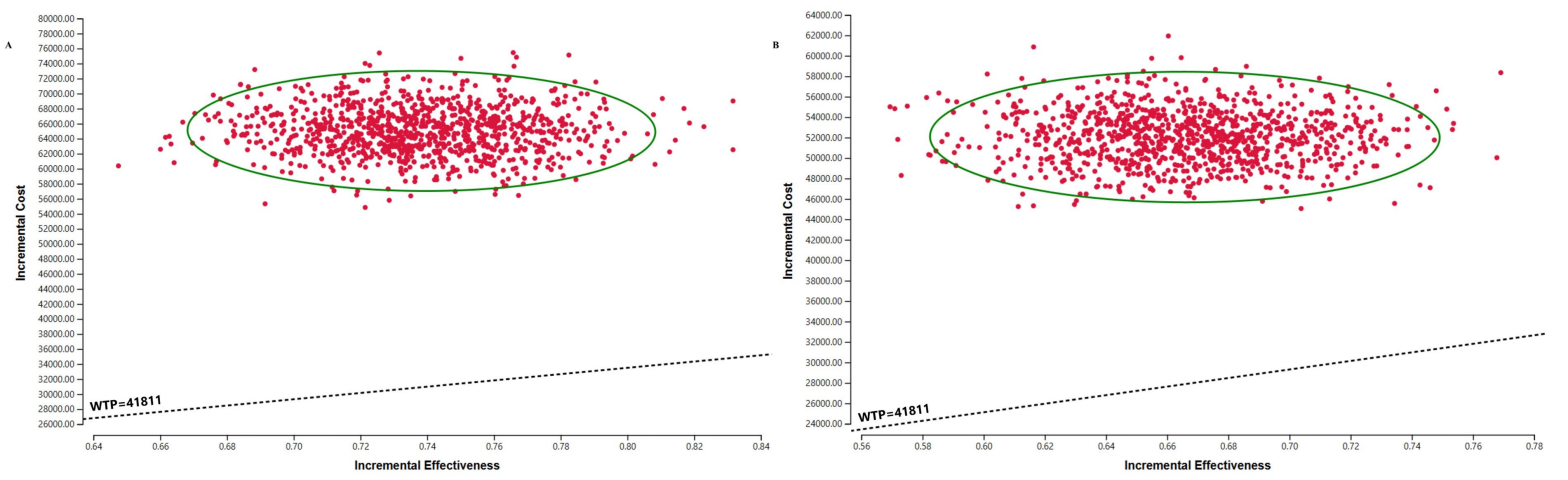
Incremental cost-effectiveness scatter plot of sac-TMT versus platinum- based chemotherapy. **(A)** PSM. **(B)** Markov model. The black dashed lines indicate the WTP threshold.

**Figure 4:**
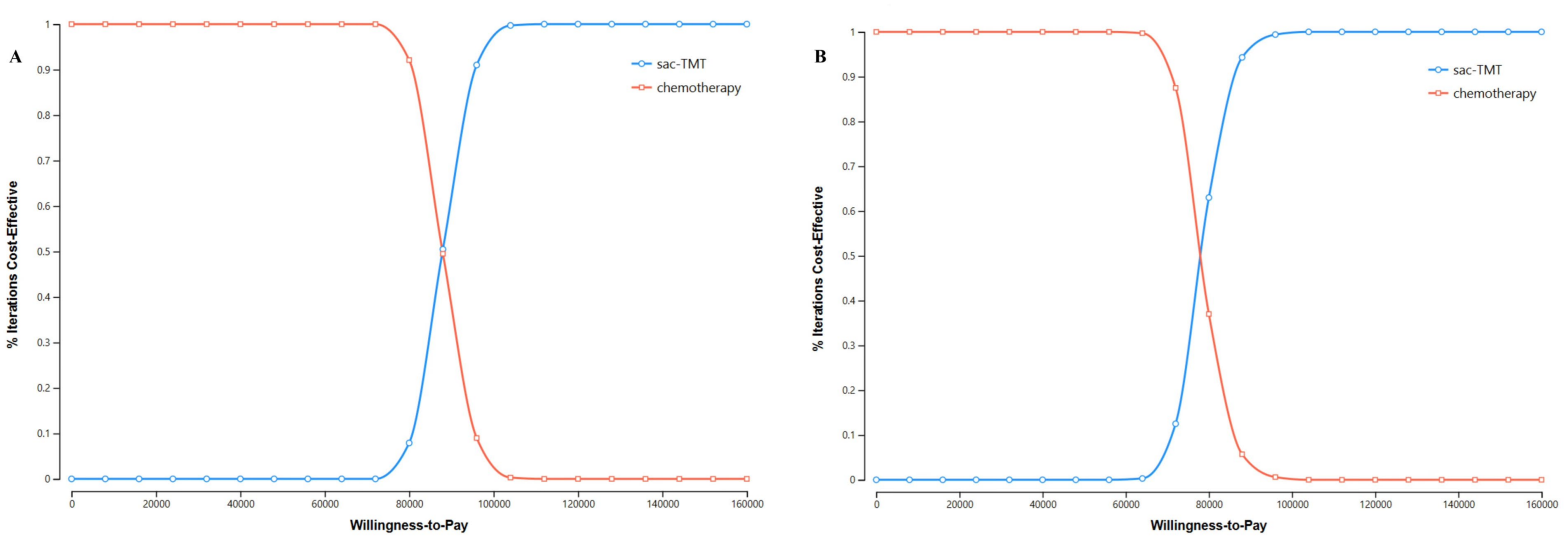
Cost-effectiveness acceptability curve of sac-TMT versus platinum-based chemotherapy. **(A)** PSM. **(B)** Markov model.

The results of the PSA are presented in the incremental cost-effectiveness scatter plot (Figure 3) and the CEAC (Figure 4). As shown in Figure 3, the ICERs for both models fell in the first quadrant and lay above the WTP threshold line, indicating that the probability of the sac-TMT regimen being cost-effective was 0%. Figure 4 shows that in the PSA, the probability of sac-TMT being cost-effective increased gradually as the WTP threshold rose. When the WTP threshold exceeded $80,000 per QALY, sac-TMT became more likely to be cost-effective than chemotherapy. Notably, the sensitivity analysis results from the Markov model were similar to those obtained from the PSM.

### Scenario analysis

**Figure 5:**
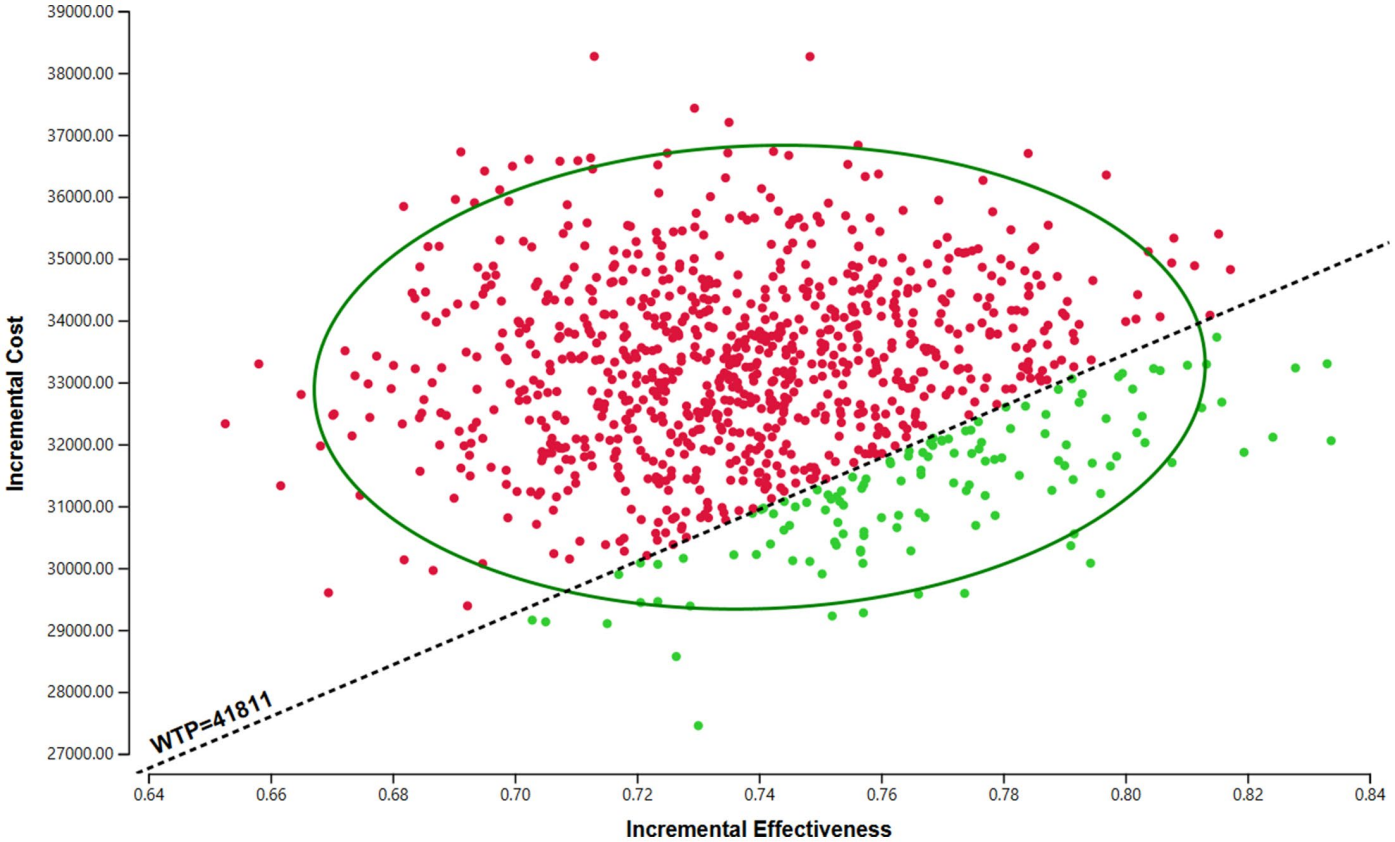
Incremental Cost-Effectiveness Scatter Plot for Scenario 1 (PSM). The results of the PSM and Markov models are consistent.

Scenario Analysis 1 (Table 3, Figure 5): Changes in drug price affect only the total cost of the treatment strategy and do not alter QALYs. As the drug price decreases, the ICER exhibits a marked downward trend. Table 3 shows that the ICER of sac-TMT was $44,760.43/QALY in the Part SA model and $39,318.36/QALY in the Markov model. The results were directionally consistent across both models. Figure 5 shows that the probability of sac-TMT being cost-effective was 13.1% when priced at its current non-reimbursed cost. Therefore, the drug would only become economically viable after inclusion in the national reimbursement list with appropriate coverage policies.

**Table 3:** Base-case results of scenario analysis 1.

Model	Treatment	Total cost	∆ Cost	QALYs	∆ QALYs	ICER ($/QALY)
PSM	Chemotherapy	8930.73	—	0.59	—	—
	sac-TMT	41995.44	33064.71	1.32	0.74	44760.43
Markov	Chemotherapy	10715.65	—	0.6	—	—
	sac-TMT	36921.68	26206.03	1.26	0.67	39318.36

**Figure 6:**
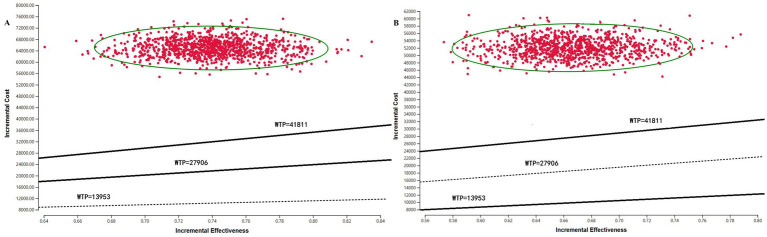
Incremental Cost-Effectiveness Scatter Plot for Scenario Analysis 2. **(A)** PSM. **(B)** Markov model.

Scenario Analysis 2 (Figure 6): The WTP threshold is widely used to evaluate the economic affordability of innovative drugs. The WTP range adopted in this study balances disease severity with social resource allocation, thereby improving the rationality of the economic evaluation. The ICERs for both models fell in the first quadrant and lay above the WTP threshold line, indicating that the probability of cost-effectiveness was 0.

**Figure 7:**
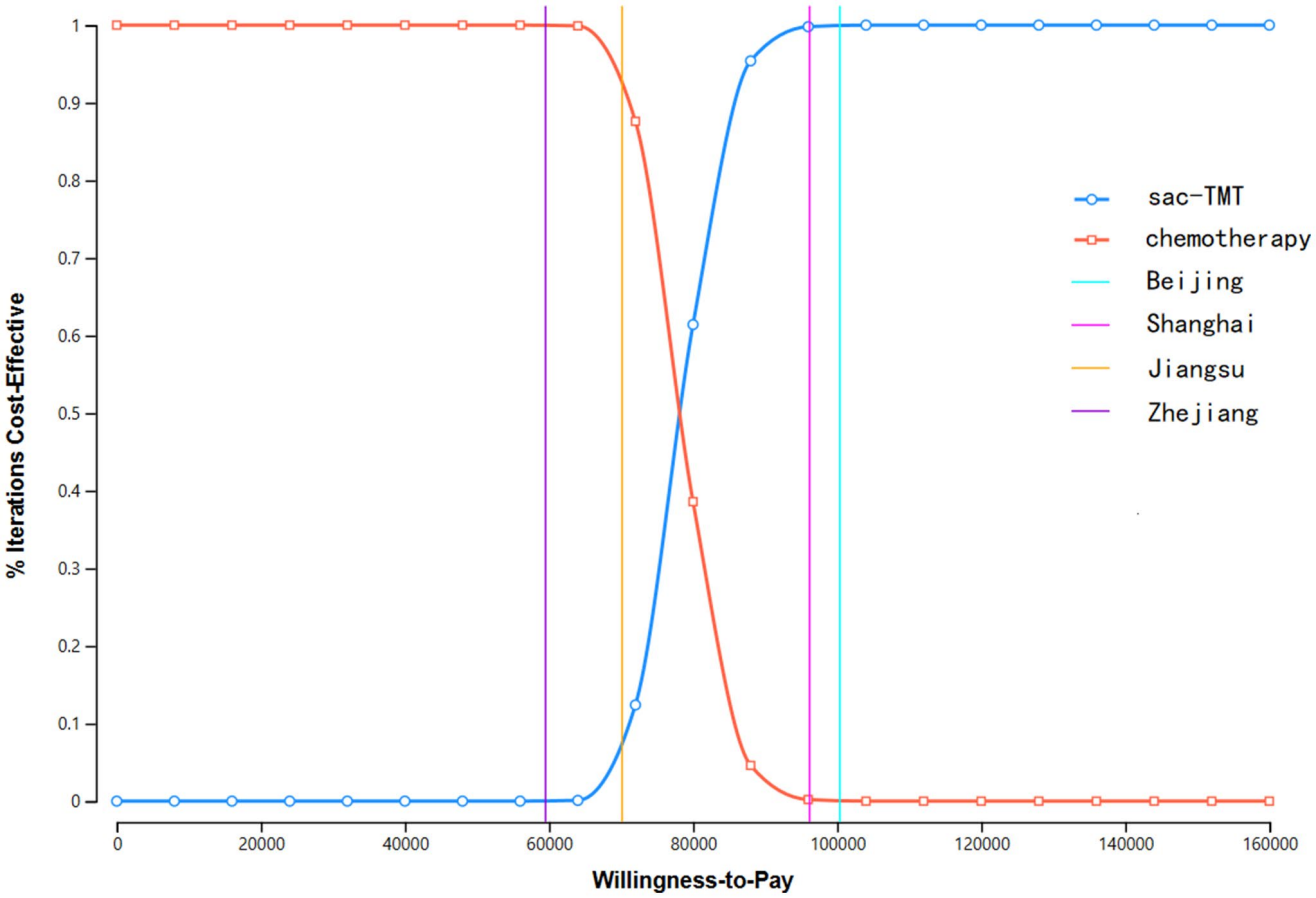
Cost-Effectiveness Acceptance Curve (CECA) for Scenario Analysis 2 (PSM). The purple line represents the WTP threshold for Zhejiang Province, the yellow line denotes the hospital payment threshold for Jiangsu Province, The pink line represents the WTP threshold for Shanghai, The blue line represents the WTP threshold for Beijing.

Scenario Analysis 3 (Figure 7): According to the latest data released by the National Bureau of Statistics of China (8), substantial disparities exist in per capita gross domestic product across different regions of China. When the willingness-to-pay threshold was set at three times the per capita GDP of Beijing (100,380 USD/QALY) or Shanghai (96,180 USD/QALY), the probability that the sac-TMT regimen was cost-effective reached 100%. In contrast, when the threshold was set at three times the per capita GDP of Jiangsu (70,155 USD/QALY) or Zhejiang (59,532 USD/QALY), this probability decreased to 0%.

## Discussion

This economic evaluation demonstrates that, from the perspective of the Chinese healthcare system, sac-TMT is not a cost-effective treatment option compared to platinum-based chemotherapy for patients with advanced EGFR-mutant NSCLC following prior EGFR-TKI therapy. In the PSM, sac-TMT led to an ICER of $87,922.33/QALY. The Markov model showed similar trends: ICER $78,131.64/QALY—both far above the WTP threshold. Notably, PSA revealed a 0% probability of sac-TMT being cost-effective under the current threshold. However, scenario analysis suggested that including sac-TMT in medical insurance or raising the payment threshold could make cost-effectiveness feasible (up to 100% probability).

In cost-effectiveness evaluation, the choice of simulation model remains a core controversial issue (19). Economic models are inherently subject to uncertainty. Although sensitivity analysis can address parameter-level uncertainty, the commonly used PSM and Markov model in oncology pharmacoeconomic evaluations often fail to fully account for structural uncertainty arising from model selection. The construction of traditional Markov models requires additional assumptions, such as whether patients in the PFS and PD states can transition directly to death. In contrast, the partitioned survival model is based on clinical trial survival data and does not require additional estimation of transition probabilities, leading to fundamental differences in modeling logic between the two approaches. Several studies have explored the applicability of these two models. McEwan et al. (20) found that the partitioned survival model could accurately reproduce observed survival outcomes, whereas Coly et al. (21) argued that it introduces inherent bias and tends to favor disease progression-related treatment strategies. Goeree et al. (22) demonstrated that core outcomes, such as the ICER, calculated by the two models were generally consistent. However, Williams et al. (23) identified obvious discrepancies in predictive results, confirming objective differences between the modeling approaches. Therefore, adopting multi-model cross-validation in pharmacoeconomic assessments is an effective strategy to improve the reliability and robustness of research conclusions.

Current debates regarding the WTP threshold in oncology focus on whether universal thresholds adequately reflect the unique value attributes of cancer conditions. The standardized, homogeneous setting of general WTP thresholds markedly conflicts with the inherent heterogeneity of malignant diseases. The commonly adopted domestic WTP range of 1–3 times per capita GDP lacks population-based empirical evidence from China and fails to account for variations in disease burden across different tumor types, while unified criteria for disease-specific WTP thresholds have yet to be established. Existing evidence confirms that WTP benchmarks should differ across disease categories (24). On the one hand, the pricing of antitumor agents is substantially higher than that of conventional medications; on the other hand, patients with cancer have urgent demands for prolonged survival and improved quality of life, accompanied by a higher willingness to pay. Accordingly, scholars have proposed setting elevated WTP thresholds specifically for oncology drugs. For instance, in the United States, the general WTP threshold for common medicines ranges from USD 50,000 to 100,000 per QALY, whereas a higher threshold of USD 100,000 to 150,000 per QALY is applied to antitumor therapies (25). Furthermore, universal WTP thresholds neglect the substantial unmet medical needs in oncology, which may lead to conservative cost-effectiveness judgments for high-cost regimens—such as sacituzumab govitecan—that provide substantial survival benefits. In response to these concerns, this study adopted WTP thresholds of 1-fold, 2-fold, and 3-fold per capita GDP in scenario analyses, aiming to explore differentiated tumor-specific thresholds and enable a more accurate evaluation of the clinical and economic value of novel antitumor drugs.

Nevertheless, this unified GDP-based threshold has limitations in evaluating oncology interventions. It is based on the overall socioeconomic level and fails to reflect the higher social value of survival gains for advanced cancer patients. A universal threshold may therefore underestimate the clinical value of innovative therapies and misclassify them as not cost-effective. Unlike international practices that adopt higher thresholds for life-threatening cancers, the current threshold does not incorporate a severe disease premium, potentially biasing the evaluation. Furthermore, the threshold relies solely on single-year per capita GDP, ignoring dynamic factors such as economic growth and medical inflation (26).

Meanwhile, the economic disparities across different regions should also be considered. Regional economic disparities across China lead to geographic variations in the WTP threshold, complicating threshold selection in oncology (27). This justifies incorporating four developed cities—Beijing, Shanghai, Jiangsu, and Zhejiang—into our scenario analysis. With per capita GDP above the national average, these regions show greater affordability for innovative anti-cancer therapies and stronger insurance support for high-cost drugs. However, subtle policy differences among the four cities also result in divergent local WTP levels, reinforcing that a single universal threshold cannot accommodate regional clinical practices. Moreover, applying developed region thresholds to less developed areas may exceed local affordability and cause imbalanced resource allocation. In response, this study adopted three times the per capita GDP of each city as the localized WTP threshold, complying with domestic evaluation standards and ensuring comparability. Given ongoing debates and our scenario analysis results, we suggest that differentiated regional thresholds—based on local GDP and insurance fiscal status—together with a severe cancer premium, would improve the accuracy and applicability of cost-effectiveness conclusions for sacituzumab govitecan versus platinum-based chemotherapy. A recent cost-effectiveness analysis of trastuzumab deruxtecan (T-DXd) for HER2-mutant NSCLC serves as a relevant comparator (28). Conducted from both U.S. and Chinese healthcare system perspectives, the study by Cai et al. reported that T-DXd was not cost-effective in the U.S. versus docetaxel (ICER: $338,998/QALY) or nivolumab (ICER: $1,437,258/QALY). Similarly, in the Chinese context, T-DXd was not cost-effective compared to docetaxel (ICER: 137,959 CNY/ QALY), nivolumab (ICER: 623,806 CNY/QALY), or pyrotinib (ICER: 515,447 CNY/QALY). That analysis also identified drug price as the principal determinant of the unfavorable ICERs. Consequently, despite its clinical efficacy, T-DXd did not demonstrate a cost-effectiveness advantage in either setting. This parallel finding underscores the decisive role of pricing in determining the economic feasibility of novel, effective ADCs such as T-DXd and, by extension, sac-TMT. Therefore, to translate the full clinical and societal value of such innovative therapies into practice, it is essential that pricing and reimbursement negotiations—led by agencies such as China’s National Healthcare Security Administration—rigorously consider affordability and align drug prices with the health outcomes they deliver.

Several limitations should be considered when interpreting the findings of this study. First, the model inputs were primarily derived from published data from the OptiTROP-Lung04 trial and supplemental literature. Detailed information regarding post-progression treatment sequences and trial-based, patient-reported quality-of-life data were not fully accessible. Consequently, necessary assumptions were made based on current clinical guidelines and published utility studies. Although these assumptions are clinically reasonable, they may introduce uncertainty into the model outcomes. Second, to preserve model parsimony, only grade ≥3 TRAEs with an absolute incidence difference of ≥5% between the trial arms were incorporated into the cost analysis. Although sensitivity analysis indicated that variations in TRAE costs did not substantially influence the ICER, this simplification may have resulted in a marginal underestimation of total treatment costs. Third, the OS data for sac-TMT were immature at the trial’s data cutoff, with the median OS not yet reached. Long-term survival was extrapolated using standard parametric survival models, a conventional yet inherently uncertain methodological approach in oncology cost-effectiveness analyses. Although the optimal distribution was selected based on statistical fit criteria, this reliance on extrapolation from immature survival data increases the uncertainty surrounding long-term outcome projections.

## Conclusion

In conclusion, within the Chinese healthcare system, sac-TMT does not demonstrate cost-effectiveness compared with platinum-based chemotherapy for EGFR-mutant NSCLC in the absence of drug price adjustments or targeted medical insurance policies. The scenario analysis conducted in this study can inform medical insurance reimbursement decisions for this agent and serve as a reference for subsequent drug pricing negotiations.

## Data Availability

The original contributions presented in the study are included in the article/Supplementary material, further inquiries can be directed to the corresponding author.

## Supplementary material

The Supplementary material for this article can befound online at: https://www.frontiersin.org/articles/10.3389/fpubh.2026.1802471/full#supplementary-material
