# Supplementary material for: Cost-effectiveness analysis based on two models of sacituzumab tirumotecan versus platinum-based chemotherapy as second-line treatment in EGFR-mutant non-small-cell lung cancer
Source: Front Public Health. 2026 May 7;14:1802471. doi: 10.3389/fpubh.2026.1802471 (PMC13190414; doi:10.3389/fpubh.2026.1802471)
Supplement: Supplementary file 1 [file Data_Sheet_1.docx]

**Supplementary material**

Supplementary Table S1 Comparison of Median Reconstruction of Survival Curves

|  | **Survival indicators** | **Median time / months（95CI%）** | |
| --- | --- | --- | --- |
|  |  | **Original curve** | **Reconstructed curve curve** |
| sac-TMT | PFS | 8.3（6.7~9.9） | 8.3（6.8~9.9） |
|  | OS | NR（21.5~NE） | NA（21.5~NA） |
| chemotherapy | PFS | 4.3（4.2~5.5） | 4.4（4.2~5.5） |
|  | OS | 17.4（15.7~20.4） | 17.5（15.8~20.5） |

Supplementary Table S2 Goodness-of-Fit Statistics for Parametric Distributions

|  | **sac-TMT** | | | | | **chemotherapy** | | | | |
| --- | --- | --- | --- | --- | --- | --- | --- | --- | --- | --- |
|  | **PFS** | | **OS** | | | **PFS** | | | **OS** | |
|  | **AIC** | **BIC** | **AIC** | **BIC** | **AIC** | | **BIC** | **AIC** | | **BIC** |
| Weibull (PH) | 980.88 | 987.35 | 637.81 | 644.28 | 869.40 | | 875.87 | 846.05 | | 852.52 |
| Log-normal | 976.01 | 982.48 | 636.87 | 643.35 | 851.38 | | 857.86 | 851.44 | | 857.91 |
| Log-logistic | 978.33 | 984.81 | 636.68 | 643.16 | 848.94 | | 855.41 | 848.56 | | 855.04 |
| Gompertz | 987.67 | 994.14 | 641.32 | 647.80 | 896.72 | | 903.19 | 848.76 | | 855.23 |
| Gengamma | 977.26 | 986.97 | 638.34 | 648.05 | 852.90 | | 862.61 | 847.96 | | 857.67 |
| Gamma | 978.63 | 985.10 | 637.18 | 643.65 | 858.41 | | 864.89 | 846.56 | | 853.03 |
| Exponential | 989.85 | 993.09 | 642.71 | 645.95 | 905.72 | | 908.96 | 866.80 | | 870.04 |


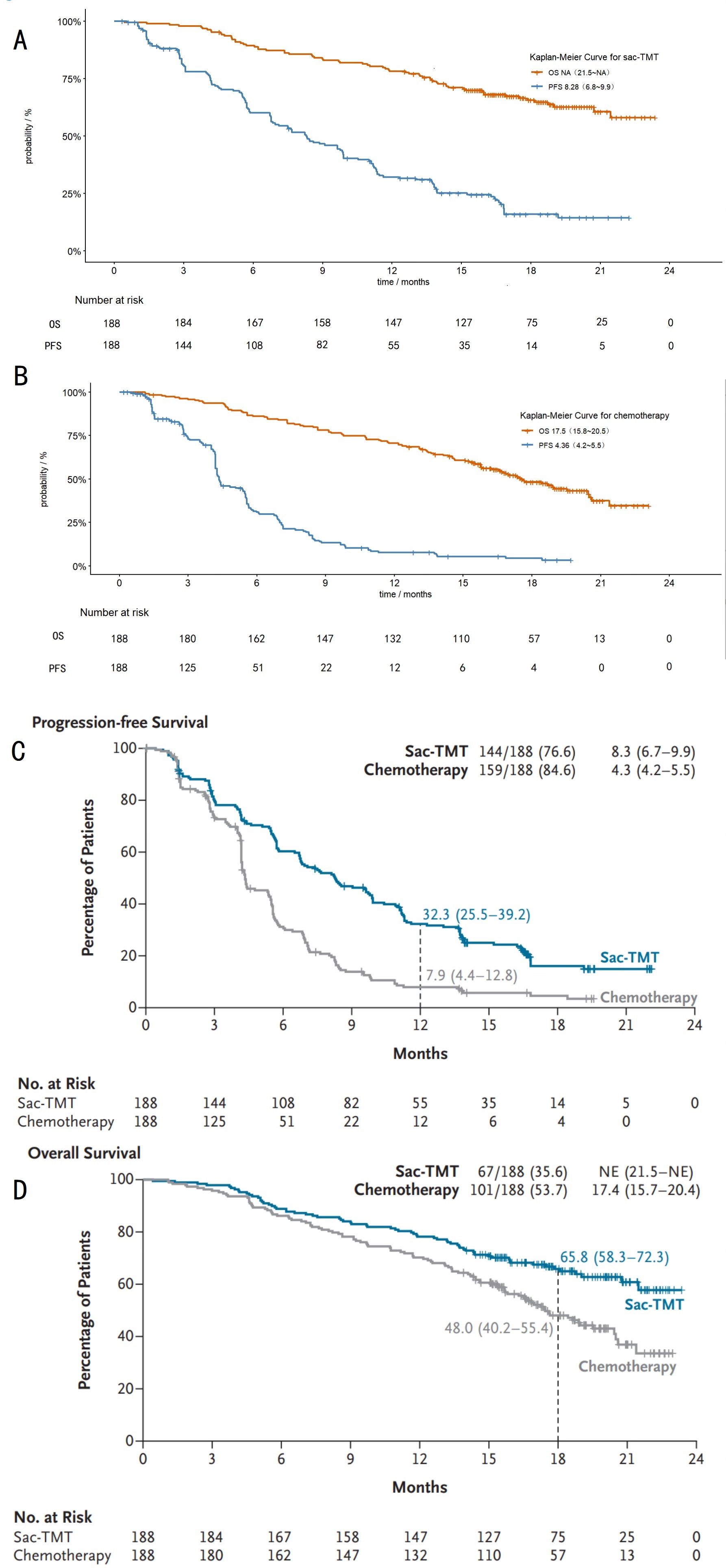


Supplementary Figure S1 Validation of Digitized Kaplan-Meier Curves for PFS and OS Against the Original Published Trial Data
Panel A shows the Kaplan-Meier survival curves for the sac-TMT group.

Panel B shows the Kaplan-Meier survival curves for the chemotherapy group.

Panel C shows the PFS curves comparing the sac-TMT group and the chemotherapy group.

Panel D shows the OS curves comparing the sac-TMT group and the chemotherapy group.
